# Supplementary material for: Exposure to graphene oxide sheets alters the expression of reference genes used for real-time RT-qPCR normalization
Source: Sci Rep. 2019 Aug 29;9:12520. doi: 10.1038/s41598-019-48970-z (PMC6715796; doi:10.1038/s41598-019-48970-z)
Supplement: Supplementary file 1 — Supplementary Information [file 41598_2019_48970_MOESM1_ESM.pdf]

SUPPORTING INFORMATION

Exposure to graphene oxide sheets alters the expression of reference genes used for real-time RT-qPCR normalization

Irene de Lázaro[1,2,*] and Kostas Kostarelos[1]

[1] Nanomedicine Lab, Faculty Biology, Medicine and Health and National Graphene Institute, AV Hill Building, The University of Manchester, Manchester M13 9PT, United Kingdom

[2] Present address: John A. Paulson School of Engineering and Applied Sciences, Harvard University, Cambridge, MA, 02138, USA; Wyss Institute for Biologically Inspired Engineering, Harvard University, Boston, MA, 02115, USA

* Correspondence to: idelazaro@g.harvard.edu

Supporting Tables

Table S1. Physicochemical characterization of GO material used in this study.

	Technique	*Results*
Lateral dimensions*	*AFM*	0.050 – 0.5 µm
	TEM	0.1 – 2 µm
Thickness*	*AFM*	1.4 ± 0.5 nm (1-2 layers)
Optical Properties	*Absorbance (λ = 230 nm)*	A = 0.050 * C_{GO} (µg/mL)
	Fluorescence (λ_{exc} = 525 nm)	F_{600} = 0.823 * C_{GO} (µg/mL)
Degree of defects (I_D/I_G)	*Raman spectroscopy*	1.36 ± 0.03
Surface charge	*ζ-potential*	-55.9 ± 1.4 mV
Functionalisation degree	*TGA*	41%
Chemical composition (Purity)	*XPS*	C: 67.6%, O: 32.2%, (99.8%)<br>S: 0.2%
C:O ratio	*XPS*	2.1
π-π, O=C-O, C=O,<br>C-O-C, C-C & C=C	*XPS*	3.0%, 15.3%, 31.2%,<br>11.3%, 39.2%

*Lateral dimensions and thickness are reported as a range between the minimum and maximum sizes detected. Full characterization of the material is provided in Mukherjee et al[15].

Table S2. MIQE checklist.

See separate XLS file

Table S3. RNA yield and integrity assessment, MCF7 samples.

Sample	[RNA] µg/ml	$A_{260/280}$	$A_{260/230}$	28S/18S	RIN
Untreated 1	554	2.01	2.1	3.0	9.2
Untreated 2	583	2.06	2.02	2.4	9.2
Untreated 3	535	1.87	1.93	3.2	9.5
5 µg/ml 1	559	2.02	2.08	3.8	9.3
5 µg/ml 2	639	2.04	2.18	2.5	9.6
5 µg/ml 3	563	2.02	1.82	2.8	9.3
10 µg/ml 1	636	2.02	1.99	3.7	9.4
10 µg/ml 2	597	2.01	2.11	3.3	9.4
10 µg/ml 3	723	2.03	2.19	3.9	9.4
50 µg/ml 1	523	1.79	1.70	3.0	9.6
50 µg/ml 2	594	2.02	1.75	3.0	9.8
50 µg/ml 3	529	1.99	2.09	2.7	9.8

[RNA] µg/ml, $A_{260/A280}$ and $A_{260/230}$ are calculated by spectroscopy; 28S/18S ratios and RIN numbers are calculated upon RNA denaturation and electrophoretic separation **(Figure S1, a)**. The table shows the results corresponding to all four experimental groups, n=3.

Table S4. RNA yield and integrity assessment, MEF samples.

Sample	[RNA] µg/ml	A260/280	A260/230	28S/18S	RIN
Untreated 1	358	1.89	1.88	3.1	9.1
Untreated 2	333	1.96	1.93	2.8	9.3
Untreated 3	324	2.01	2.19	2.7	9.3
5 µg/ml 1	266	2.09	1.71	2.2	9.6
5 µg/ml 2	223	2.03	2.1	2.5	8.9
5 µg/ml 3	246	1.99	2.14	2.4	9.1
10 µg/ml 1	371	1.82	1.95	2.5	9.3
10 µg/ml 2	263	1.77	1.77	2.1	9.3
10 µg/ml 3	252	1.92	1.77	2.3	9.3
50 µg/ml 1	96	1.96	2.04	2.0	9.4
50 µg/ml 2	176	1.99	2.09	2.4	9.2
50 µg/ml 3	110	1.71	1.75	2.6	9.3

[RNA] µg/ml, $A_{260/A280}$ and $A_{260/230}$ are calculated by spectroscopy; 28S/18S ratios and RIN numbers are calculated upon RNA denaturation and electrophoretic separation **(Figure S1, b)**. The table shows the results corresponding to all four experimental groups, n=3.

Table S5. Details of primer pairs used in MCF7 study (human).

Gene symbol	Accession number (mRNA)	Primer sequences	Amplicon size (bp)	E	R^2
**RPS13**	NM_001017.2	**Fwd** CGCTCTCCTTTCGTTGCCT<br>**Rv** CGCTGCGTCGATAGGGTAAA	96	2.11	0.9956
**RPL27**	NM_000988.3	**Fwd** ATCGCCAAGAGATCAAAGATAA<br>**Rv** TCTGAAGACATCCTTATTGACG	123	2.02	0.9993
**RPL30**	NM_000989.3	**Fwd** ACAGCATGCGGAAAATACTAC<br>Rv AAAGGAAAATTTTGCAGGTTT	158	1.99	0.9987
**OAZ1**	NM_004152.3<br>NM_001301020.1	**Fwd** CTCCACTGCTGTAGTAACCCG<br>Rv GATCCCTCTGACTATTCCCTCG	104	1.75	0.9998
**ACTB**	NM_001101.3	**Fwd** AGCACAGAGCCTCGCCTTT<br>**Rv** GAGCGCGGCGATATCATCA	82	2.0	0.9961
**GAPDH**	NM_001289746.1<br>NM_001289745.1<br>NM_001256799.2<br>NM_002046.5	**Fwd** CCACATGGCCTCCAAGGAGTAAGAC<br>**Rv** AGGAGGGGAGATTCAGTGTGGTGGG	131	2.0	0.9984
**MAPK1**	NM_002745.4<br>NM_138957.3	**Fwd** TCCCAAATGCTGACTCCAAAG<br>**Rv** CATGTCGAACTTGAATGGTGC	164	2.08	0.9993
**UBC**	NM_021009.6	**Fwd** GCCTTAGAACCCCAGTATCAG<br>**Rv** AAGAAAACCAGTGCCCTAGAG	74	1.98	0.9907
**HMBS**	NM_000190.3<br>NM_001024382.1<br>NM_001258208.1<br>NM_001258209.1<br>XM_017017629.1<br>XM_005271531.1<br>XM_005271532.1<br>XM_005271533.3<br>XM_011542796.1	**Fwd** AGCTTGCTCGCATACAGACG<br>**Rv** AGCTCCTTGGTAAACAGGCTT	157	2.03	0.9990
**TBP**	NM_003194.4<br>NM_001172085.1	**Fwd** CCACTCACAGACTCTCACAAC<br>**Rv** CTGCGGTACAATCCCAGAACT	127	2.00	0.9968

E, efficiency of qPCR reaction; R^2, coefficient of determination from linear regression of Cq values (cDNA serial dilution). Primer pairs amplify all transcription variants with equal amplicon length.

Table S6. Details of primer pairs used in MEF study (mouse).

Gene symbol	Accession number (mRNA)	Primer sequences	Amplicon size (bp)	E	R^2
**Rps13**	NM_026533.3	**Fwd** CCCAGGTCCGTTTTGTGACT<br>**Rv** TCCTCTCAAGGTGCTTTCGG	132	2.01	0.9972
**Rpl27**	NM_011289.3	**Fwd** CAAAAACGCAGTGCCCGA<br>**Rv** CTTACGGAGAGGTGGCTTCA	120	2.27	0.9907
**Rpl30**	NM_009083.4<br>NM_001163485.1	**Fwd** GAAGAGCTTTGCATTGTGGGAG<br>**Rv** CCATCTTCCTGCCTTAGGTGC	102	1.95	0.9948
**OAZ1**	NM_008753.4<br>NM_001301034.1	**Fwd** GGGTTGCCCTTAATTGCTGT<br>**Rv** TCTTGTCGTTAGACGTCGGC	187	1.99	0.9985
**Actb**	NM_007393.5	**Fwd** CTGAGCTGCGTTTTACACCC<br>**Rv** CGCCTTCACCGTTCCAGTTT	200	1.93	0.9995
**Gapdh**	NM_001289726.1<br>NM_008084.3<br>XM_017321385.1	**Fwd** AGGTCGGTGTGAACGGATTTG<br>**Rv** TGTAGACCATGTAGTTGAGGTCA	123	2.00	0.9988
**Mapk1**	NM_011949.3<br>NM_001038663.1<br>XM_006522147.3	**Fwd** GGTTGTTCCCAAATGCTGACT<br>**Rv** CAACTTCAATCCTCTTGTGAGGG	84	1.99	0.9979
**Ubc**	NM_019639.4	**Fwd** CAAACAGGAAGACAGACGTACC<br>**Rv** CCCATCACACCCAAGAACAAG	80	2.04	0.9986
**Hmbs**	NM_013551.2<br>NM_001110251.1	**Fwd** ATCTTGGACCTAGTGAGTGTGT<br>**Rv** GTACAGTTGCCCATCTTTCATCA	141	2.01	0.9985
**Tbp**	NM_013684.3	**Fwd** TTTGGCTAGGTTTCTGCGGT<br>**Rv** GCCCTGAGCATAAGGTGGAA	195	2.04	0.9984

E, efficiency of qPCR reaction; R^2, coefficient of determination from linear regression of Cq values (cDNA serial dilution). Primer pairs amplify all transcription variants with equal amplicon length.

Supporting Figures

Supporting Figure 1

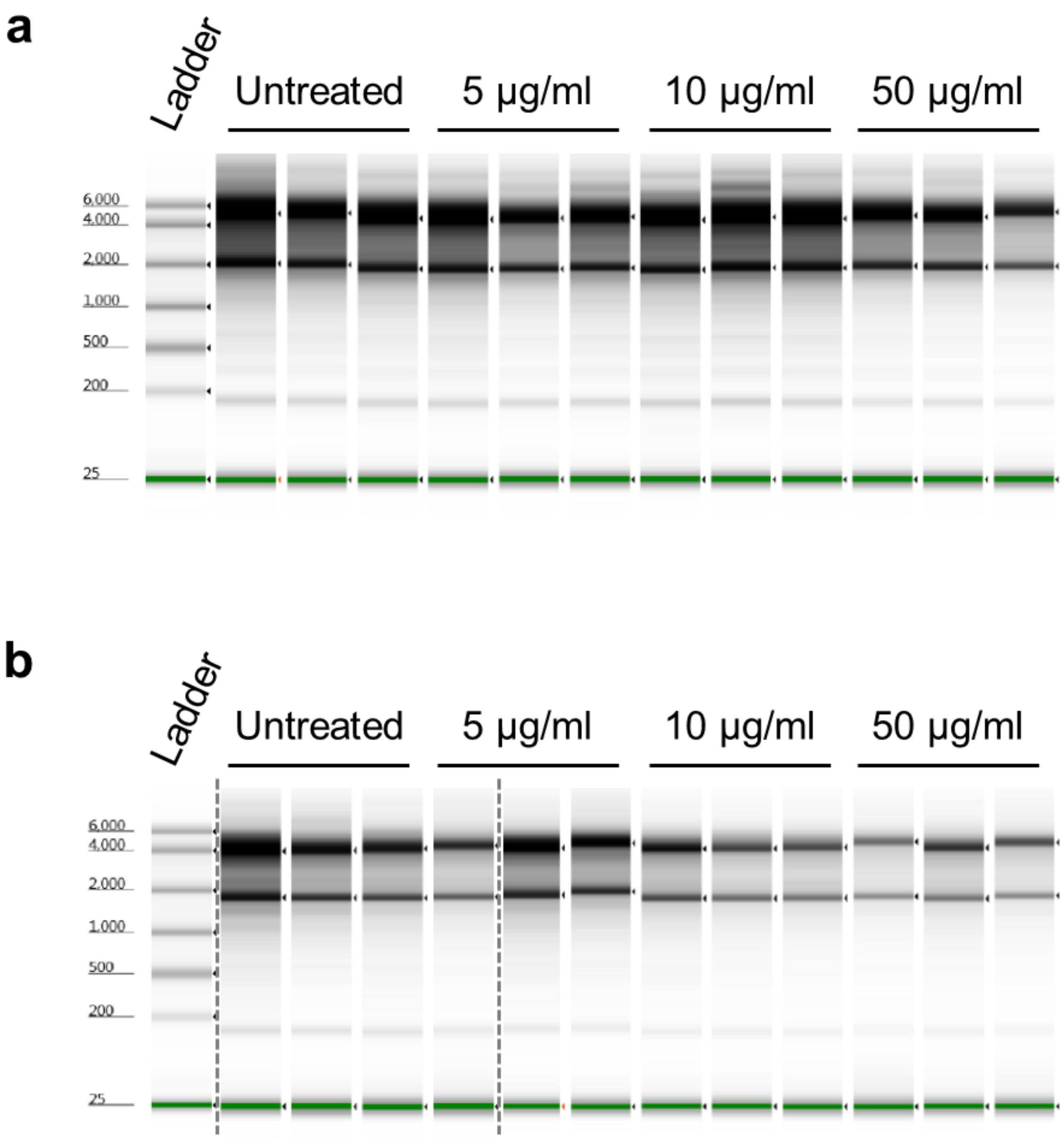

Figure S1. Analysis of RNA integrity on the 2200 TapeStation system. Gel electrophoresis shows the separation profile of individual **(a)** MCF7 and **(b)** MEF samples, including 28S, 18S, small rRNAs and lower marker. RIN numbers and 28S/18S ratios are provided in **Tables S3 and S4**. When gels have been cropped to organize the samples in consecutive order, cropping sites are delineated by grey dashed lines.

Supporting Figure 2

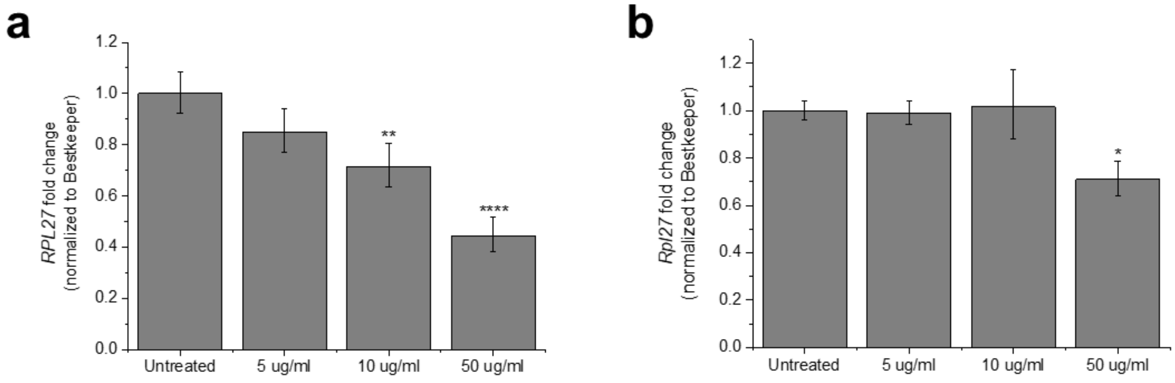

Figure S2. *RPL27* mRNA levels normalized to Bestkeeper index. (a) *RPL27* expression in MCF7 cells exposed to GO, normalized to Bestkeeper index. **(b)** *Rpl27* expression in MEFs exposed to GO, normalized to Bestkeeper index. Bars represent fold change; error bars represent propagation of standard error (SE). *p<0.05, **p<0.01 and ****p<0.0001, assessed by one-way ANOVA and Tukey's test, n=3.

Supporting Figure 3

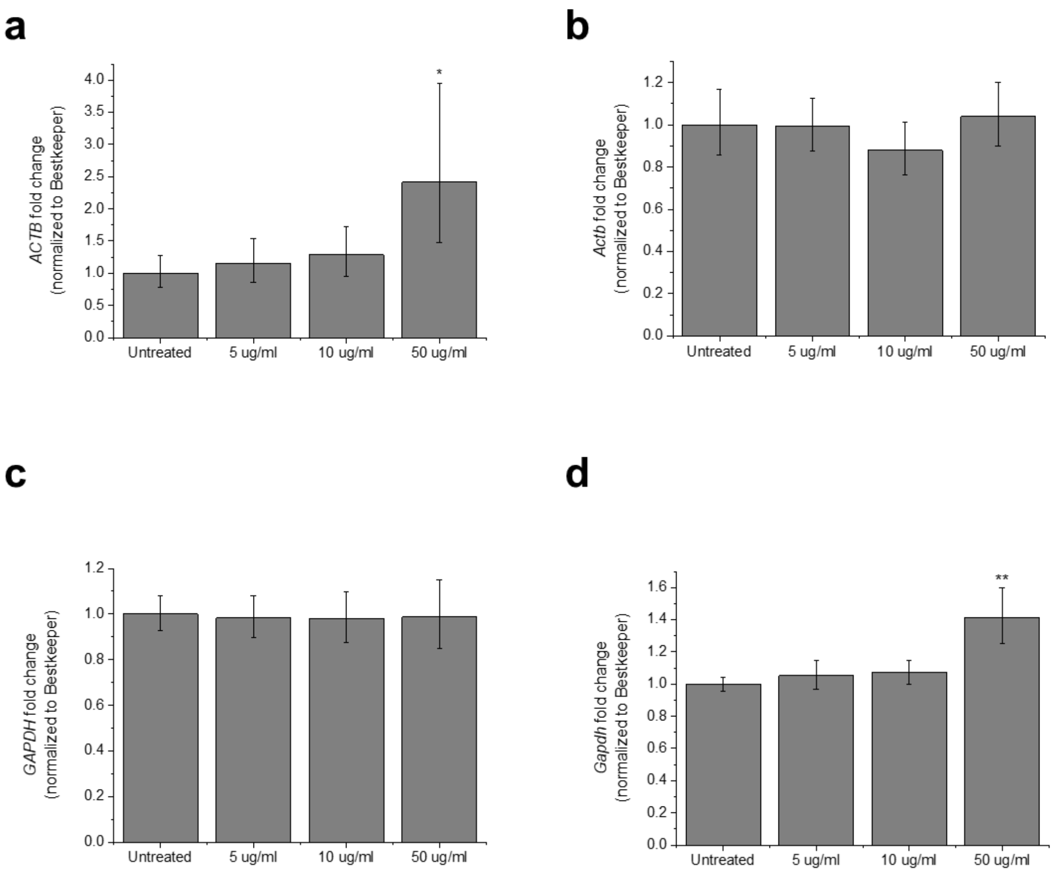

Figure S3. *ACTB* and *Gapdh* mRNA levels normalized to Bestkeeper index. (a) *ACTB* and **(b)** *GAPDH* expression in MCF7 cells exposed to GO, normalized to Bestkeeper index. **(c)** *Actb* and **(d)** *Gapdh* expression in MEFs exposed to GO, normalized to Bestkeeper index. Bars represent fold change; error bars represent propagation of standard error (SE). *$p<0.05$ and **$p<0.01$, assessed by one-way ANOVA and Tukey's test, n=3.
